# Outage Performance Analysis of Relay Selection Schemes in Wireless Energy Harvesting Cooperative Networks over Non-Identical Rayleigh Fading Channels †

**DOI:** 10.3390/s16030295

**Published:** 2016-02-26

**Authors:** Nhu Tri Do, Vo Nguyen Quoc Bao, Beongku An

**Affiliations:** 1 Department of Electronics and Computer Engineering in Graduate School, Hongik University, Sejong 30016, Korea; dotrinhu@gmail.com; 2 Department of Wireless Communications, Posts and Telecommunications Institute of Technology, Ho Chi Minh City 710372, Vietnam; baovnq@ptithcm.edu.vn; 3 Department of Computer and Information Communications Engineering, Hongik University, Sejong 30016, Korea

**Keywords:** cooperative networks, decode-and-forward, wireless energy harvesting, best relay selection, power splitting, i.n.i.d. Rayleigh fading

## Abstract

In this paper, we study relay selection in decode-and-forward wireless energy harvesting cooperative networks. In contrast to conventional cooperative networks, the relays harvest energy from the source’s radio-frequency radiation and then use that energy to forward the source information. Considering power splitting receiver architecture used at relays to harvest energy, we are concerned with the performance of two popular relay selection schemes, namely, partial relay selection (PRS) scheme and optimal relay selection (ORS) scheme. In particular, we analyze the system performance in terms of outage probability (OP) over independent and non-identical (i.n.i.d.) Rayleigh fading channels. We derive the closed-form approximations for the system outage probabilities of both schemes and validate the analysis by the Monte-Carlo simulation. The numerical results provide comprehensive performance comparison between the PRS and ORS schemes and reveal the effect of wireless energy harvesting on the outage performances of both schemes. Additionally, we also show the advantages and drawbacks of the wireless energy harvesting cooperative networks and compare to the conventional cooperative networks.

## 1. Introduction

Energy harvesting from natural resources is unstable since it mainly depends on environment conditions. Thus, it is less attractive to apply into wireless communications networks where terminals are required to be potable and stable. This difficulty motivates the concept of wireless energy harvesting (WEH) which makes mobiles scavenge energy from electromagnetic waves. Specifically, the simultaneous wireless information and power transfer (SWIPT) has more attraction among several wireless energy harvesting techniques. The fundamental idea that lies behind SWIPT is that the receiver can harvest energy and decode information from radio signals [1]. Following the pioneering work, later practical designs for SWIPT receiver architecture have separated the received signal into two orthogonal parts in the domains of time, power, antenna, or space [2]. More specifically, two well-known receiver architectures for SWIPT, namely, time-switching and power-switching, have been intensively studied (see, e.g., [3,4] and the following related works).

On the other hand, cooperative communications using relays have been recognized as an efficient way to extend coverage of wireless networks. Two relaying protocols commonly used for cooperative networks are amplify-and-forward (AF) and decode-and-forward (DF) [5]. In the AF protocol, the relay first amplifies the source signals and then sends the amplified signal to the destination. In the DF protocol, the relay decodes the source signal and then sends the representation of the source signal to the destination. Once there are multiple relays available, relay selection protocols, e.g., partial relay selection [6] and optimal relay selection [7], have been proposed to assist the transmission. Recently, energy harvesting has been showed as an attractive solution to prolong the operation of cooperative networks. By using energy harvesting node as relay, the network life-time can be significantly improved [8].

Different from the conventional cooperative networks, operation of WEH cooperative networks depends on the amount of energy that the relay harvests from the source. More specifically, in order to establish a cooperative transmission between a source-destination pair, the source transmits both energy and information simultaneously to the relay. The relay then uses the harvested energy (but not its own energy) to send the source information to the destination. Thus, performance of the relaying system mainly depends on the energy harvesting process at the relay when the source-destination direct link is not available.

Several works in the literature have studied wireless energy harvesting using RF signal for classical cooperative networks which consist of one source, one destination, and one relay. The authors in [9] investigated a relaying network in which nodes harvest energy in a different fashion: the download links (*i.e.*, access point-source, access point-relay links) are used for wireless energy transfer and the upload link (*i.e.*, source-relay, relay-access point links) are used for wireless information transfer. In [10], the authors investigated a greedy switching policy of whether a relay can transmit or not for a three-node relay network, where the relay harvests energy from source signal. Considering the same three-node relay network using AF protocol, the authors in [11] investigated the throughput performance of two proposed protocols, namely, time switching-based relaying (TSR) protocol and power switching-based relaying (PSR) protocol. Later in [12], the throughput and the ergodic capacity of TSR and PSR protocols are analyzed for a DF cooperative network with one wireless energy harvesting relay. In [13], the authors considered a full-duplex cooperative system, where the relay harvests energy using time-switching architecture and forwards the source signal using either AF or DF protocols.

Assuming that there are multiple available relays that assist the source-destination transmission, relay selection schemes have studied in wireless energy harvesting cooperative networks. In our previous work [14], the PRS scheme was investigated in wireless energy harvesting relay networks over independent and identical (i.i.d.) Rayleigh fading channels. The closed-form approximation expression of the OP was derived. The work in [15] used linear program to determine the optimal transmission scheme for a dual-hop relay system with multiple energy harvesting relays in order to minimize the transmission time for a given packet. Assuming that location of relays follows a homogeneous Poisson point process, the authors in [16] investigated three relay selection schemes in the cases of one and multiple sources. Considering the application of WEH to underlay cognitive radio relaying network, the partial relay selection was investigated in [17]. The outage performance of both time switching and power splitting receiver architecture were analyzed. On the other hand, the optimal relay selection scheme for underlay cognitive WEH relay network was studied in [18]. Very recently, in [19], relay selection schemes based on battery information of the wireless energy harvesting relays were investigated.

Different from the above related works, this paper considers dual-hop DF cooperative networks with multiple WEH relays over independent and non-identical (i.n.i.d.) Rayleigh fading channels, where the direct link between source and destination is not available due to severe fading or shadowing. We use the power splitting receiver (PSR) mechanism [3] for the relays to harvest energy from the source RF radiation. The main contributions of the paper are summarized as follows:

- We are concerned with the application of wireless energy harvesting to cooperative networks. We analyze the performance two popular relay selection schemes, namely, partial relay selection (PRS) and optimal relay selection (ORS) schemes in wireless energy harvesting cooperative networks under independent and non-identical Rayleigh fading channels. To the best of authors’ knowledge, the study that takes into account both these two schemes together in such fading environment has not been carried out in the literature.
- We evaluate the performance of those schemes in terms of outage probability (OP) over the independent and non-identical Rayleigh fading channels. Moreover, we provide the new closed-form approximation for the outage probabilities achieved by PRS and ORS schemes, respectively, which have not been appeared in the literature. To the best of our knowledge, the integrals in the final outage probabilities, *i.e.*, Equations (20) and (28), respectively, do not have the closed-form expressions for the case of the limits of the integral that we face against in the paper. In order to solve this issue, we used the Maclaurin series to derive the approximation and then examined the convergence of the approximation.
- We then provide numerical results in order to show the impacts of system parameters, such as power splitting ratio, relays’ position, target data rate on the outage performance of the system. In addition, in our simulation the relays are randomly distributed between the source and the destination which makes the simulation is more realistic compared to the previous works, e.g., [17,18]. On the other hand, the performance comparisons between PRS and ORS schemes are discussed to point out the advantages as well as the drawbacks of each schemes. Additionally, we compare the diversity gains of cooperative networks with and without using wireless energy harvesting technology.

The rest of the paper is arranged as follows. Section 2 introduces the system model and presents the partial relay selection and optimal relay selection schemes. The outage analysis is carried out in Section 3. Numerical results are shown in Section 4. Finally, the conclusions are given in Section 5.

## 2. System Model and Relay Selection Schemes

### 2.1. System Description

**Figure 1:**
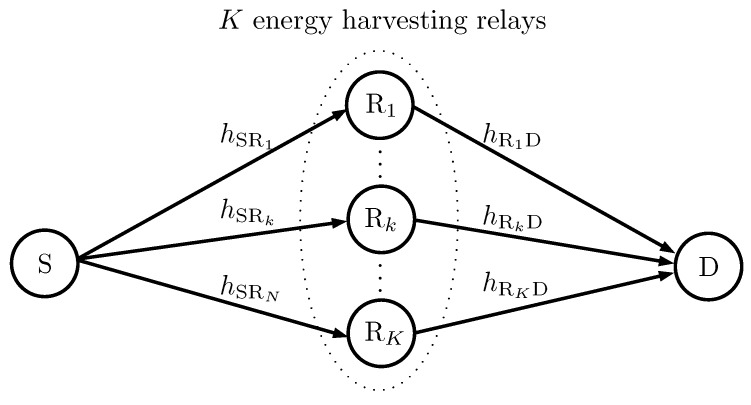
A dual-hop decode-and-forward cooperative network with *K* energy harvesting relays.

Let us consider a decode-and-forward cooperative network with one source (S), one destination (D), and *K* energy harvesting relays denoted as $R_{k}$ with k=1,2,…,K as shown in Figure 1. For notational convenience, let $R=\{R_{k}|k=1,2,\ldots ,K\}$ denote the set of the cooperative relays. All nodes are equipped with single antenna and operate in a common frequency band in half-duplex mode. The source is considered as an energy unconstrained node and transmits with a constant transmit power $P_{S}$. The direct link between the source and the destination is assumed to be not available due to the severe shadowing and path-loss.

**Figure 2:**
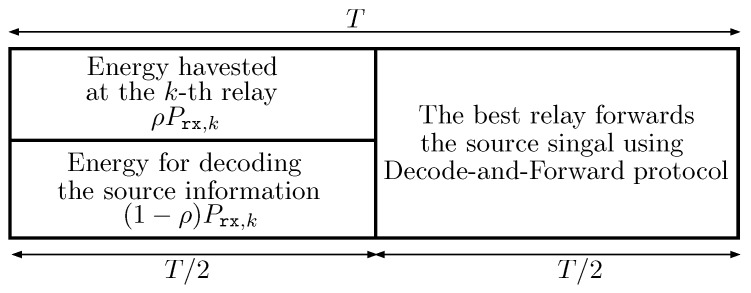
The time slot structure of power splitting mechanism for energy harvesting and signal forwarding at the relays using decode-and-forward protocol.

Since the source-destination link is not available, we consider the use of cooperative relays and the decode-and-forward protocol at the relays to assist the source-destination transmission. More specifically, a DF relaying transmission between S and D is carried out in each fixed block time *T* and is divided into two phases. We assume perfect synchronization in the network, but how to achieve this synchronization is beyond the scope of this paper. In the first phase of T/2 time, the source broadcasts its signals. The cooperative relays listen and harvest energy from the source signals using power splitting receiver architecture. Figure 2 depicts the time slot structure of the power splitting receiver mechanism for harvesting energy and information forwarding. By using the power splitting technique, the relay divides the received signal into two streams with the splitting ratio *ρ*, where 0<ρ<1. The stream with power of $\rho P_{\mathrm{rx},R_{k}}$ is used for energy harvesting, where $P_{\mathrm{rx},R_{k}}$ denotes the received power from the source during the first phase at the *k*-th relay. The remaining stream with power of $\left(1-\rho \right)P_{\mathrm{rx},R_{k}}$ is used for information decoding [2].

In the second phase of T/2 time, only the best relay among *K* available cooperative relays is selected by either partial relay selection (PRS) or optimal relay selection (ORS) schemes to forward the re-encoded version of the received source signal to the destination. Using PRS scheme, the best relay is selected based on the channel state information (CSI) of one of the two hops, *i.e.*, source-relay or relay-destination hops. In contrast, the ORS scheme chooses the best relay which provides the best end-to-end path between source and destination. Different from the PRS and ORS schemes that have been studied in the conventional cooperative networks, the transmit power of the best relay in wireless powered cooperative networks depends on the energy that it harvests from the source signal. As a result, the performance of the relay selection schemes depends on the energy harvesting strategy at the relays in which the power slitting ratio *ρ* is the main parameter. It is noticed that in our system model, we assume that the source signal is the only source that provides energy for the relays and the harvested energy is the only source of transmit power of the relays.

Additionally, we assume that all wireless links exhibit frequency non-selective Rayleigh block fading, *i.e.*, channel coefficients are constant during one block time *T*. The independent and identical distribution (i.i.d.) assumption is often used in performance analysis of cooperative networks, e.g., in [9,11,13,14,17]. In this paper, we however investigate the relay selection schemes over independent and non-identical (i.n.i.d.) Rayleigh fading channels which is more closely to practical wireless networks.

A question raised is how relay selection schemes chooses the best relay among available relays. In a centralized design, there is a coordinator which collects the instantaneous channel state information in the networks. It then makes the the decision on the selected relay based on a certain criteria (see, e.g., [20,21] and the references therein). In decentralized fashion, since there is no coordinator point choosing the best relay, the destination, for example as in [7], broadcasts a clear-to-send (CTS) packet to trigger relays to start their count-down time to transmit. Based on certain criteria (e.g., the distance from relays to the destination), the best relay which has smallest count-down time will seize the channel. In this paper, we focus on investigating the application of wireless energy to data transmission while the energy spent for sending control messages is negligible.

It is noticed that the PRS scheme selects the optimal relay based on the channel state information (CSI) of the channel from source to relays while the ORS scheme takes into account the CSI of both source-relays and relays-destination channels. It is pointed out that the ORS scheme increases the cooperative overhead compared to the PRS scheme [16]. As we mentioned above, the effects of overhead on system performance is beyond the scope of this paper. In line with the related works, we here assume that the CSI is available at any terminals in the network. On the other hand, the PSR scheme takes into account the impact of the energy harvesting process on the selection of the best relay. As described in Equation (2), the energy harvesting process at each relay takes into account only the channel gain of the first hop. The CSI of the second hop does not involve in the energy harvesting process. Based on the criteria described in Equation (8), the best relay is also the relay that harvests the highest energy from the source signal during the first phase. Therefore, the PRS scheme can choose the best relay with the highest harvested energy while the ORS scheme does not point out the harvested energy information of the chosen relay.

In the following, we first present the signal modeling of the the DF relaying protocol and the energy harvesting process, and then the PRS and ORS scheme are mathematically presented.

### 2.2. Signal Modeling

The received signal at the relay $R_{k}$ from the source S is given by

(1) $$y_{{\mathrm{SR}}_{k}}=\sqrt{P_{S}}h_{{\mathrm{SR}}_{k}}s+w_{a,R_{k}}$$

where $h_{{\mathrm{SR}}_{k}}$ is the fading coefficient of the channel from S to $R_{k}$, *s* denotes the transmitted signal from the source and we assume that $E\{|s{|}^{2}\}=1$, where E{·} is the statistical expectation operator, |·| is the absolute value operator, $w_{a,R_{k}}∼\mathrm{CN}\left(0,\sigma_{a,R_{k}}^{2}\right)$ is the additive white Gaussian noise (AWGN) caused by the antenna of the *k*-th relay.

Considering power splitting receiver mechanism, $\sqrt{\rho }y_{{\mathrm{SR}}_{k}}$ is used for harvesting energy, the remaining received signal $\sqrt{1-\rho }y_{{\mathrm{SR}}_{k}}$ is used for decoding the source information. Here, *ρ* denotes the power splitting ratio. In this paper, we want to focus on investigating the performance of relay selection schemes in wireless energy harvesting cooperative networks instead of the performance of the wireless energy harvesting process. Therefore, for the sake of simplicity, we assume that all relays have the same power splitting ratios. This mechanism is called static power splitting [3,22]. Thus, the harvested energy during the first phase at relay $R_{k}$ can be expressed as [11]

(2) $$E_{R_{k}}=\eta \rho P_{S}g_{{\mathrm{SR}}_{k}}T/2$$

where 0<η<1 is the energy conversion efficiency, and $g_{{\mathrm{SR}}_{k}}≜{\left|h_{{\mathrm{SR}}_{k}}\right|}^{2}$ is the channel gain of the channel between S and $R_{k}$.

The remaining received signal stream is converted into baseband signal for decoding the source information. The received baseband signal at the relay $R_{k}$ is given by

(3) $$\begin{array}{cc} y_{{\mathrm{SR}}_{k}}^{\mathrm{base}} & =\sqrt{1-\rho }y_{{\mathrm{SR}}_{k}}\\ & =\sqrt{\left(1-\rho \right)}\left(\sqrt{P_{S}}h_{{\mathrm{SR}}_{k}}s+w_{a,R_{k}}\right)+w_{c,R_{k}} \end{array}$$

where $y_{{\mathrm{SR}}_{k}}^{\mathrm{base}}$ denotes the received baseband signal, $w_{c,R_{k}}∼\mathrm{CN}\left(0,\sigma_{c,R_{k}}^{2}\right)$ is the AWGN caused by the process of downconverting signal from passband to baseband [3]. Recalling that the received signal at the wireless energy harvesting relay is affected by two kinds of noise, *i.e.*, one is created by the antenna of the relay, and the other one is created by signal conversion process [3]. Each kind of noise has different impact on the performance of the harvesting energy process [11]. However, we are not going to investigate the impact of the noises at the receiver on the OP of the considered schemes in this paper.

From Equation (3), the instantaneous signal-to-noise ratio (SNR) at the relay $R_{k}$ is given by

(4) $$\begin{array}{cc} \gamma_{{\mathrm{SR}}_{k}} & =\frac{\left(1-\rho \right)P_{S}g_{{\mathrm{SR}}_{k}}}{\left(1-\rho \right)\sigma_{a,R_{k}}^{2}+\sigma_{c,R_{k}}^{2}}\\ & =\Psi_{R}g_{{\mathrm{SR}}_{k}} \end{array}$$

where $\Psi_{R}=\left(\left(1-\rho \right)P_{S}\right)/\left(\left(1-\rho \right)\sigma_{R}^{2}+\sigma_{R}^{2}\right)$, and for the sake of simplicity, we assume that all relays have the same noise variance, *i.e.*, $\sigma_{a,R_{k}}^{2}=\sigma_{c,R_{k}}^{2}=\sigma_{R}^{2}$.

Considering the DF protocol, the relay decodes the source signal and then forwards the re-encoded signal to the destination. Since all the energy harvested during the first phase is used to forward the source signal in the second phase of T/2 time, using Equation (2), the transmit power of $R_{k}$ is given by

(5) $$\begin{array}{cc} P_{R_{k}} & =\frac{E_{R_{k}}}{T/2}\\ & =\eta \rho P_{S}g_{{\mathrm{SR}}_{k}} \end{array}$$

The received signal at D from $R_{k}$ is given by

(6) $$y_{R_{k}D}=\sqrt{P_{R_{k}}}h_{R_{k}D}\bar{s}+w_{D}$$

where $h_{R_{k}D}$ is the fading coefficient of the channel from $R_{k}$ to D, $\bar{s}$ is the re-encoded version of *s*, and $w_{D}∼\mathrm{CN}\left(0,\sigma_{D}^{2}\right)$ is the AWGN at D.

From Equations (5) and (6), the instantaneous SNR of the channel between $R_{k}$ and D is given by

(7) $$\begin{array}{cc} \gamma_{R_{k}D} & =\frac{\eta \rho P_{S}g_{{\mathrm{SR}}_{k}}g_{R_{k}D}}{\sigma_{D}^{2}}\\ & =\Psi_{D}g_{{\mathrm{SR}}_{k}}g_{R_{k}D} \end{array}$$

where $\Psi_{D}=\eta \rho P_{S}/\sigma_{D}^{2}$.

### 2.3. Relay Selection Schemes

Aiming at improving the system performance, we here consider relay selection schemes in order to choose the best relay that assist the transmission between S and D. In this paper, two relay selection schemes, namely, partial relay selection scheme (PRS) and optimal relay selection scheme (ORS), are considered.

#### 2.3.1. Partial Relay Selection (PRS) Scheme

In PRS scheme, the relay that has the highest instantaneous SNR of the channel from S to $R_{k}$ will be selected as the best relay, and it will assist the source-destination transmission. Mathematically, the best relay, denoted as $R_{b}^{\mathrm{PRS}}$, where b∈R, is chosen as

(8) $$R_{b}^{\mathrm{PRS}}=\arg\max_{k\in R}\gamma_{{\mathrm{SR}}_{k}}$$

where R represents the set of relays, *i.e.*, $R≜\{R_{k}|k=1,2,...,N$}.

#### 2.3.2. Optimal Relay Selection (ORS) Scheme

In DF protocol, the end-to-end transmission is in failure if one of the two hops is corrupted. Thus, the end-to-end SNR of the path from S via $R_{k}$ to D is given by

(9) $$\gamma_{e2e,k}=\min\left(\gamma_{{\mathrm{SR}}_{k}},\gamma_{R_{k}D}\right)$$

The ORS scheme takes into account the instantaneous SNR of both the link from S to $R_{k}$ and the link from $R_{k}$ to D. More specifically, the relay that has the highest end-to-end instantaneous SNR is viewed as the optimal relay. Mathematically, the best relay, denoted as $R_{b}^{\mathrm{ORS}}$, where b∈R, is chosen as

(10) $$R_{b}^{\mathrm{ORS}}=\arg\max_{k\in R}\gamma_{e2e,k}$$

## 3. Performance Analysis

In this section, we will study the performance of the considered schemes in terms of outage probability over i.n.i.d. Rayleigh fading channels. The outage probability of the system is defined as the probability that the system capacity is below a fixed transmission rate, $R_{th}$ in bits/sec/Hz [23]. Note that, throughout this paper, the notation “OP” stands for outage probability but does not for outage performance.

Assuming that all wireless channels in the network exhibit Rayleigh fading, the channel gains, $g_{ij}≜{\left|h_{ij}\right|}^{2}$, are independent and exponential random variables with means of $\lambda_{ij}$, where $i\in S≜\{S,R_{k}\}$ and $j\in D≜\{R_{k},D\}$. Therefore, the corresponding cumulative distribution function (CDF) and probability density function (CDF) of $g_{ij}$ are given by

(11) $$\begin{array}{cc} F_{g_{ij}}\left(z\right) & =1-e^{-\frac{z}{\lambda_{ij}}} \end{array}$$

(12) $$\begin{array}{cc} f_{g_{ij}}\left(z\right) & =\frac{1}{\lambda_{ij}}e^{-\frac{z}{\lambda_{ij}}} \end{array}$$

respectively, where $\lambda_{ij}=d_{ij}^{-\beta }$ is the average channel gain, $d_{ij}$ is the Euclid distance between node *i* and node *j*, *β* is the path-loss exponent.

### 3.1. Outage Performance of the PRS Scheme

The capacity of the channel from S to $R_{b}^{\mathrm{PRS}}$, and from $R_{b}^{\mathrm{PRS}}$ to D is given by

(13) $$C_{{\mathrm{SR}}_{b}}^{\mathrm{PRS}}=\frac{1}{2}\log_{2}\left(1+\gamma_{{\mathrm{SR}}_{b}}^{\mathrm{PRS}}\right),\;C_{R_{b}D}^{\mathrm{PRS}}=\frac{1}{2}\log_{2}\left(1+\gamma_{R_{b}D}^{\mathrm{PRS}}\right)$$

respectively, where the factor 1/2 appears because we consider a dual-hop transmission, $\gamma_{{\mathrm{SR}}_{b}}^{\mathrm{PRS}}\;≜\;\max_{k\in R}\gamma_{{\mathrm{SR}}_{k}}$ represents the SNR of the channel from the source to the best relay which is selected by the PRS scheme, and $\gamma_{R_{b}D}^{\mathrm{PRS}}$ represents the SNR of the channel from the best relay to the destination and is treated as $\gamma_{R_{k}D}$.

In dual-hop DF transmission, the failure of one of the two hops leads to the failure of the transmission. Thus, the outage probability of the PRS scheme, $P_{\mathrm{out}}^{\mathrm{PRS}}$, is mathematically written as

(14) $$P_{\mathrm{out}}^{\mathrm{PRS}}=\mathrm{Pr}\left(C_{{\mathrm{SR}}_{b}}^{\mathrm{PRS}}<R_{th}\right)+\mathrm{Pr}\left(C_{{\mathrm{SR}}_{b}}^{\mathrm{PRS}}\geq R_{th},C_{R_{b}D}^{\mathrm{PRS}}<R_{th}\right)$$

**Lemma 1.**  *Let $g_{{\mathrm{SR}}_{b}}^{\mathrm{PRS}}≜\max_{k\in R}g_{{\mathrm{SR}}_{k}}$ denote the instantaneous channel gain of the channel from the source to the best relay that selected by the PRS scheme. The cumulative distribution function (CDF) and probability density function (PDF) of $g_{{\mathrm{SR}}_{b}}^{\mathrm{PRS}}$ are given as*

(15) $$\begin{array}{cc} F_{g_{{\mathrm{SR}}_{b}}^{\mathrm{PRS}}}\left(z\right) & =1-\sum_{k=1}^{K}{\left(-1\right)}^{k-1}\underset{n_{1}<\cdots <n_{k}}{\sum_{n_{1}=1}^{K}\cdots \sum_{n_{k}=1}^{K}}e^{-x\sum_{t=1}^{k}\frac{1}{\lambda_{{\mathrm{SR}}_{n_{t}}}}} \end{array}$$

(16) $$\begin{array}{cc} f_{g_{{\mathrm{SR}}_{b}}^{\mathrm{PRS}}}\left(z\right) & =\sum_{k=1}^{K}{\left(-1\right)}^{k-1}\underset{n_{1}<\cdots <n_{k}}{\sum_{n_{1}=1}^{K}\cdots \sum_{n_{k}=1}^{K}}\sum_{t=1}^{k}\frac{1}{\lambda_{{\mathrm{SR}}_{n_{t}}}}e^{-x\sum_{t=1}^{k}\frac{1}{\lambda_{{\mathrm{SR}}_{n_{t}}}}} \end{array}$$

*respectively.*

**Proof.**  See Appendix A. ☐

Using Equations (4) and (7), the OP of the PRS scheme is further expressed as

(17) $$\begin{array}{cc} P_{\mathrm{out}}^{\mathrm{PRS}} & =\mathrm{Pr}\left(\gamma_{{\mathrm{SR}}_{b}}^{\mathrm{PRS}}<\gamma_{th}\right)+\mathrm{Pr}\left(\gamma_{{\mathrm{SR}}_{b}}^{\mathrm{PRS}}\geq \gamma_{th},\gamma_{R_{b}D}^{\mathrm{PRS}}<\gamma_{th}\right)\\ & =\underset{I_{1}}{\underset{︸}{\mathrm{Pr}(\Psi_{R}g_{{\mathrm{SR}}_{b}}^{\mathrm{PRS}}<\gamma_{th})}}+\underset{I_{2}}{\underset{︸}{\mathrm{Pr}(\Psi_{R}g_{{\mathrm{SR}}_{b}}^{\mathrm{PRS}}\geq \gamma_{th},\Psi_{D}g_{{\mathrm{SR}}_{b}}^{\mathrm{PRS}}g_{R_{b}D}^{\mathrm{PRS}}<\gamma_{th})}} \end{array}$$

where $\gamma_{th}≜2^{2R_{th}}-1$ denotes the SNR threshold for correctly decoding information.

By using Equation (15) in Lemma 1, $I_{1}$ is obtained as

(18) $$\begin{array}{cc} I_{1} & =\mathrm{Pr}\left(g_{{\mathrm{SR}}_{b}}^{\mathrm{PRS}}<\frac{\gamma_{th}}{\Psi_{R}}\right)\\ & =1-\sum_{k=1}^{K}{\left(-1\right)}^{k-1}\underset{n_{1}<\cdots <n_{k}}{\sum_{n_{1}=1}^{K}\cdots \sum_{n_{k}=1}^{K}}e^{-\frac{\gamma_{th}}{\Psi_{R}}\sum_{t=1}^{k}\frac{1}{\lambda_{{\mathrm{SR}}_{n_{t}}}}} \end{array}$$

**Corollary 1.**  *By applying Lemma 1, the term $I_{2}$ in Equation (17) is obtained as*

(19) $$\begin{array}{cc} I_{2} & =\frac{1}{K}\sum_{k=1}^{K}\sum_{l=1}^{K}{\left(-1\right)}^{l-1}\underset{n_{1}<\cdots <n_{l}}{\sum_{n_{1}=1}^{K}\cdots \sum_{n_{l}=1}^{K}}e^{-\frac{\gamma_{th}}{\Psi_{R}}\sum_{t=1}^{l}\frac{1}{\lambda_{{\mathrm{SR}}_{n_{t}}}}}-\frac{1}{K}\sum_{k=1}^{K}\int_{\gamma_{th}/\Psi_{R}}^{\infty }e^{-\frac{\gamma_{th}}{\Psi_{D}\lambda_{R_{k}D}x}}\\ & \;\times \sum_{l=1}^{K}{\left(-1\right)}^{l-1}\underset{n_{1}<\cdots <n_{l}}{\sum_{n_{1}=1}^{K}\cdots \sum_{n_{l}=1}^{K}}\sum_{t=1}^{l}\frac{1}{\lambda_{{\mathrm{SR}}_{n_{t}}}}e^{-x\sum_{t=1}^{l}\frac{1}{\lambda_{{\mathrm{SR}}_{n_{t}}}}}dx \end{array}$$

**Proof.**  See Appendix B. ☐

By plugging Equations (18) and (19) into Equation (17), and after some arrangements, the outage probability $P_{\mathrm{out}}^{\mathrm{PRS}}$ is obtained as

(20) $$\begin{array}{cc} P_{\mathrm{out}}^{\mathrm{PRS}} & =1-\frac{1}{K}\sum_{k=1}^{K}\sum_{l=1}^{K}{\left(-1\right)}^{l-1}\underset{n_{1}<\cdots <n_{l}}{\sum_{n_{1}=1}^{K}\cdots \sum_{n_{l}=1}^{K}}\sum_{t=1}^{l}\frac{1}{\lambda_{{\mathrm{SR}}_{n_{t}}}}\int_{\gamma_{th}/\Psi_{R}}^{\infty }e^{-\frac{\gamma_{th}}{\Psi_{D}\lambda_{R_{k}D}x}-x\sum_{t=1}^{l}\frac{1}{\lambda_{{\mathrm{SR}}_{n_{t}}}}}dx \end{array}$$

To the best of the authors’ knowledge, an exact closed-form expression for the integral in Equation (20) is not available for the case of $0<\gamma_{th}/\Psi_{R}<\infty$. We next provide the derivation of the closed-form approximation for the outage probability of the PRS scheme.

In order to approximate Equation (20), let us consider the following integral

(21) $$A=\int_{a_{1}}^{\infty }e^{-\frac{b_{1}}{x}-c_{1}x}dx$$

where $a_{1}=\gamma_{th}/\Psi_{R}>0$, $b_{1}=\frac{\gamma_{th}}{\Psi_{D}\lambda_{R_{k}D}}$, and $c_{1}=\sum_{t=1}^{l}\frac{1}{\lambda_{{\mathrm{SR}}_{n_{t}}}}$. Note that when $a_{1}=0$, the integral Equation (21) can be easily derived by using Equation (3.324.1) in [24].

Using Maclaurin series for the term $e^{-\frac{b_{1}}{x}}$ , Equation (21) is further expressed as

(22) $$\begin{array}{cc} A & =\int_{a_{1}}^{\infty }e^{-c_{1}x}\sum_{n=0}^{\infty }\frac{{\left(-1\right)}^{n}b_{1}^{n}}{n!x^{n}}dx\\ & =\sum_{n=0}^{\infty }\frac{{\left(-1\right)}^{n}b_{1}^{n}}{n!}\int_{a_{1}}^{\infty }\frac{e^{-c_{1}x}}{x^{n}}dx\\ & =\int_{a_{1}}^{\infty }e^{-c_{1}x}dx+b_{1}\int_{a_{1}}^{\infty }\frac{e^{-c_{1}x}}{x}dx+\sum_{n=2}^{\infty }\frac{{\left(-1\right)}^{n}b_{1}^{n}}{n!}\int_{a_{1}}^{\infty }\frac{e^{-c_{1}x}}{x^{n}}dx \end{array}$$

After some manipulations, and with the help of Equation (3.353.1) in [24], Equation (21) can be obtained as

(23) $$\begin{array}{cc} A & =\frac{e^{-a_{1}c_{1}}}{c_{1}}-b_{1}\Gamma \left(0,a_{1}c_{1}\right)+\sum_{n=2}^{\infty }\frac{{\left(-1\right)}^{n}b_{1}^{n}}{n!}\left[e^{-a_{1}c_{1}}\sum_{v=1}^{n-1}\frac{\left(v-1\right)!{\left(-c_{1}\right)}^{n-v-1}}{\left(n-1\right)!a_{1}^{k}}-\frac{{\left(-c_{1}\right)}^{n-1}}{(n-1)!}\mathrm{Ei}\left(-a_{1}c_{1}\right)\right] \end{array}$$

where Γ(·,·) is the incomplete gamma function, defined in Equation (3.350.2) in [24], and Ei(·) is the exponential integral function, defined in Equation (8.211.1) in [24]. By plugging Equation (23) into Equation (20), we obtain the closed-form expression of the approximation of the PRS scheme’s outage probability.

### 3.2. Outage Performance of the ORS Scheme

The end-to-end capacity of the channel from S via $R_{b}^{\mathrm{ORS}}$ to D is given by

(24) $$C_{e2e,b}^{\mathrm{ORS}}=\frac{1}{2}\log_{2}\left(1+\gamma_{e2e,b}^{\mathrm{ORS}}\right)$$

where $\gamma_{e2e,b}^{\mathrm{ORS}}≜\max_{k\in R}\gamma_{e2e,k}$ represents the end-to-end SNR of the path from S to D via the best relay which is selected by the ORS scheme.

In order to derive the OP of the ORS scheme, we first find the CDF of the end-to-end SRN of the DF transmission.

**Lemma 2.**  *Let $\gamma_{e2e,k}=\min\left(\gamma_{{\mathrm{SR}}_{k}},\gamma_{R_{k}D}\right)$ denote the end-to-end signal-to-noise ratio of the path from the source to the destination via the k-th relay $R_{k}$. The cumulative distribution function (CDF) of $\gamma_{e2e,k}$ is given as*

(25) $$\begin{array}{cc} F_{\gamma_{e2e,k}}\left(z\right) & =1-e^{-\frac{z}{\Psi_{R}\lambda_{{\mathrm{SR}}_{k}}}-\frac{\Psi_{R}}{\Psi_{D}\lambda_{R_{k}D}}}-\frac{1}{\lambda_{R_{k}D}}\int_{0}^{\Psi_{R}/\Psi_{D}}e^{-\frac{z}{\Psi_{D}\lambda_{{\mathrm{SR}}_{k}}x}-\frac{x}{\lambda_{R_{k}D}}}dx \end{array}$$

**Proof.**  See Appendix C. ☐

For the ORS scheme, the outage probability $P_{\mathrm{out}}^{\mathrm{ORS}}$ is mathematically written as

(26) $$\begin{array}{cc} P_{\mathrm{out}}^{\mathrm{ORS}} & =\mathrm{Pr}(C_{e2e,b}^{\mathrm{ORS}}<R_{th})\\ & =\mathrm{Pr}(\gamma_{e2e,b}^{\mathrm{ORS}}<\gamma_{th}) \end{array}$$

Using the definition of the end-to-end SNR in Equation (24), and due to the fact that wireless channels are assumed to be independent, $P_{\mathrm{out}}^{\mathrm{ORS}}$ is given by

(27) $$\begin{array}{cc} P_{\mathrm{out}}^{\mathrm{ORS}} & =\mathrm{Pr}\left(\max_{k\in R}\gamma_{e2e,k}<\gamma_{th}\right)\\ & =\prod_{k=1}^{K}\mathrm{Pr}\left(\gamma_{e2e,k}<\gamma_{th}\right) \end{array}$$

Apply Lemma 2 into Equation (27), the outage probability of ORS scheme is obtained as

(28) $$P_{\mathrm{out}}^{\mathrm{ORS}}=\prod_{k=1}^{K}\left(1-e^{-\frac{\gamma_{th}}{\Psi_{R}\lambda_{{\mathrm{SR}}_{k}}}-\frac{\Psi_{R}}{\Psi_{D}\lambda_{R_{k}D}}}-\frac{1}{\lambda_{R_{k}D}}\int_{0}^{\Psi_{R}/\Psi_{D}}e^{-\frac{\gamma_{th}}{\Psi_{D}\lambda_{{\mathrm{SR}}_{k}}x}-\frac{x}{\lambda_{R_{k}D}}}dx\right)$$

To the best of authors’ knowledge, the exact closed-form expression of the integral in Equation (28) for the case of $0<\Psi_{R}/\Psi_{D}<\infty$ is not available. We next provide the derivation of the closed-form approximation for the outage probability of the ORS scheme.

In order to approximate Equation (28), we consider the following integral

(29) $$B=\int_{0}^{a_{2}}e^{-\frac{b_{2}}{x}-c_{2}x}dx$$

where $a_{2}=\Psi_{R}/\Psi_{D}<\infty$, $b_{2}=\frac{\gamma_{th}}{\Psi_{D}\lambda_{{\mathrm{SR}}_{k}}}$, $c_{2}=\frac{1}{\lambda_{R_{k}D}}$. Note that when $a_{2}\to \infty$, the integral Equation (29) can be easily derived by using Equation (3.324.1) in [24].

Using Maclaurin series for the term $e^{-c_{2}x}$ in the integral, and after some manipulations and arrangements, Equation (29) is obtained as

(30) $$\begin{array}{cc} B & =\int_{0}^{a_{2}}e^{-\frac{b_{2}}{x}}\sum_{n=0}^{\infty }\frac{{\left(-1\right)}^{n}c_{2}^{n}}{n!}x^{n}dx\\ & =\sum_{n=0}^{\infty }\frac{{\left(-1\right)}^{n}c_{2}^{n}}{n!}\int_{0}^{a_{2}}x^{n}e^{-\frac{b_{2}}{x}}dx\\ & =\sum_{n=0}^{\infty }\frac{{\left(-1\right)}^{n}c_{2}^{n}}{n!b_{2}^{-1-n}}\Gamma \left(-1-n,\frac{b_{2}}{a_{2}}\right) \end{array}$$

where Γ(·,·) is the incomplete gamma function, defined in Equation (3.350.2) in [24]. By plugging Equation (30) into Equation (28), we obtain the closed-form expression of the approximation of the ORS scheme’s outage probability.

## 4. Numerical Results

In this section, the analytical results are validated by the simulation results. In addition, through numerical results, we provide the performance comparison between PRS and ORS schemes, and examine the effect of the number of relays on the outage performance in both schemes. We then make comparison on performance of wireless energy harvesting cooperative networks and the conventional cooperative networks. We use MATLAB to run the Monte-Carlo simulation and get the simulation results.

Without loss of generality, we consider the relay network in a square of unit area. The coordinates of the source S and the destination D are S=(0,0.5) and D=(1,0.5), respectively. The set of available relays has five nodes and they are randomly distributed in the square of unit area. The coordinates of the relays are as follows: $R_{1}=\left(0.1,0.2\right)$, $R_{2}=\left(0.3,0.7\right)$, $R_{3}=\left(0.4,0.5\right)$, $R_{4}=\left(0.7,0.8\right)$, and $R_{5}\;=\;\left(0.9,0.1\right)$. In each trial, the best relay among the set R will be selected to assist the source-destination transmission.

Unless otherwise stated, we set the simulation parameters as follows: energy harvesting efficiency η=1, path loss exponent β=3. For the sake of simplicity, noise powers are set as $\sigma_{R}^{2}=\sigma_{D}^{2}=1$ as in [3,17,19].

**Figure 3:**
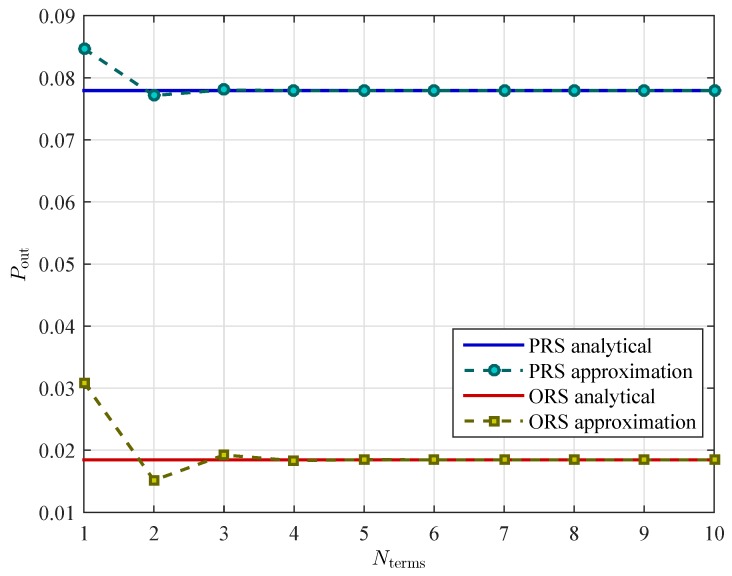
Convergence of the approximation for the outage probabilities with K=3, ρ=0.3, $P_{S}\;=\;10$ dB, and $R_{th}=2$ bits/s/Hz.

In Figure 3, we examine the convergence of the approximation for the outage probabilities of both PRS and ORS schemes. Here $N_{\mathrm{terms}}$ denotes the number of terms that we use in the Maclaurin series. As we can see from Figure 3 that the approximations quickly converge to the exact values after just few terms, *i.e.*, 4 terms.

**Figure 4:**
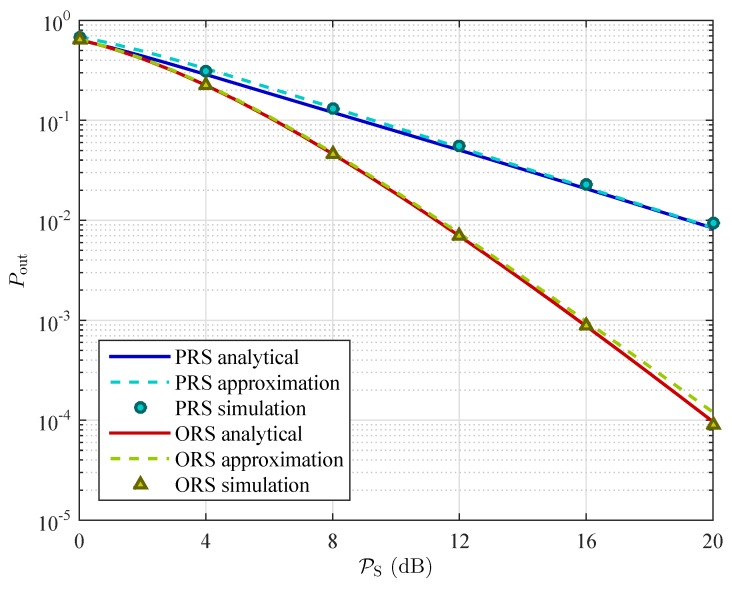
Exact analytical results, the approximation of the analytical results, and the simulation results of the outage probabilities with $N_{\mathrm{terms}}=3$, K=3, ρ=0.3, and $R_{th}=2$ bits/s/Hz.

In Figure 4, we plot the exact analytical results, the approximation of the analytical results, and the simulation results of the outage probabilities as a function of the transmit power of the source $P_{S}$. We can observe from the Figure 4 that the simulation results are well matched with the analysis results, confirming the correctness of our analysis.

**Figure 5:**
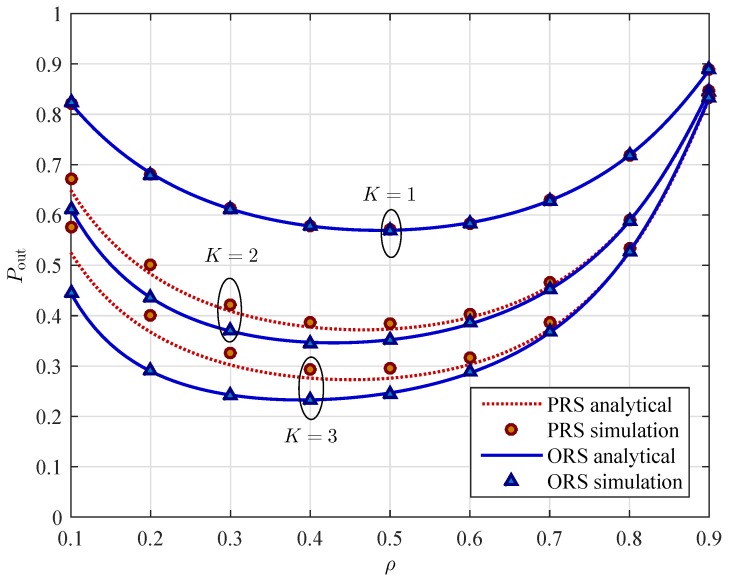
Outage probability $P_{\mathrm{out}}$ *versus* the power splitting ratio *ρ* for different values of the number of relays *K* with $P_{S}=10$ dB, and $R_{th}=3$ bits/s/Hz.

Figure 5 presents the outage probability of the two schemes as a function of the power splitting ratio *ρ*. From Figure 5, we can observe that the outage probability is a convex function with respect to the power splitting ratio ρ∈(0,1). Therefore, there is an optimal value of *ρ* for which the OP is minimum. For example, with our current parameter setting, the optimal value of *ρ* is statistically around 0.3 for both schemes. As we can see in Figure 5, the OP of both PRS and ORS schemes decreases as *ρ* increases from 0 to the optimal value and then it increases as *ρ* increases from the optimal value to 1. The reason is that increasing value of *ρ* results in the higher transmit power that the relay uses for sending the source information to the destination. Consequently, the better received SNR at the destination in the second phase is obtained and the OP of relay-destination transmission decreases. However, as the power splitting ratio *ρ* increases higher than the optimal value, the less energy is used for the relay to decode the information from the source signal. Therefore, the OP of the source-relay transmission increases. Note that the relay cannot harvest energy and decode information of the source signal at the same time. Hence, increasing power splitting ratio results in more energy can be harvested and used to forwarding signal, but less energy remains for decoding information.

**Figure 6:**
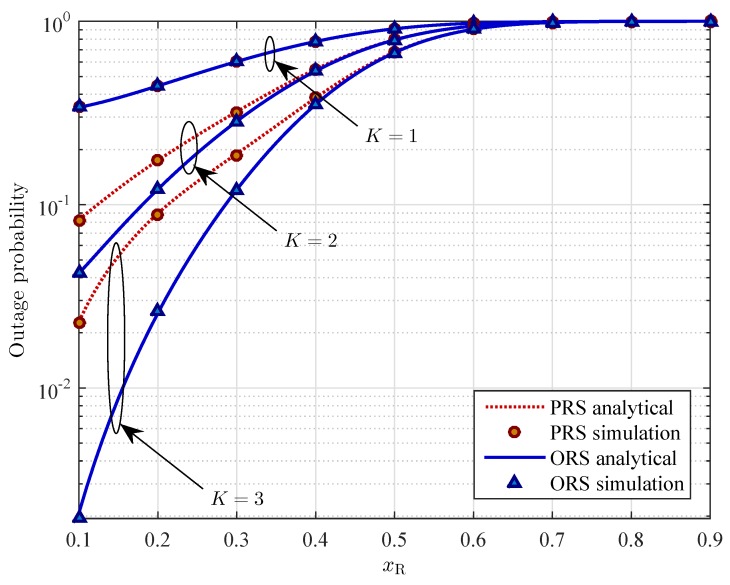
Outage probability $P_{\mathrm{out}}$ *versus* the *x*-coordinate of the relays $x_{R}$ for different values of the number of relays *K* with ρ=0.3, $P_{S}=10$ dB, and $R_{th}=3$ bits/s/Hz.

Figure 6 shows the outage probability of the two schemes as a function of *x*-coordinate of relays. In order to examine the effect of relays’ position on system performance, we choose the set of 5 relays $R=\{R_{k}|k=1,2,\ldots ,5\}$ with the same *x*-coordinates $x_{R}$, where $R_{1}=\left(x_{R},0.3\right)$, $R_{2}=\left(x_{R},0.4\right)$, $R_{3}=\left(x_{R},0.5\right)$, $R_{4}=\left(x_{R},0.6\right)$, and $R_{5}=\left(x_{R},0.7\right)$. As we can see in Figure 6, the OP increases as the relays horizontally move far away from the source and toward to the destination. This can be explained that due to the path-loss effects, as the distance between the source and the relay increases, the received power at the relay decreases, thus less energy can be harvested. On the other hand, the transmit power of the relay in the second phase depends only on the harvested energy during the first phase. Consequently, the OP of the system increases.

**Figure 7:**
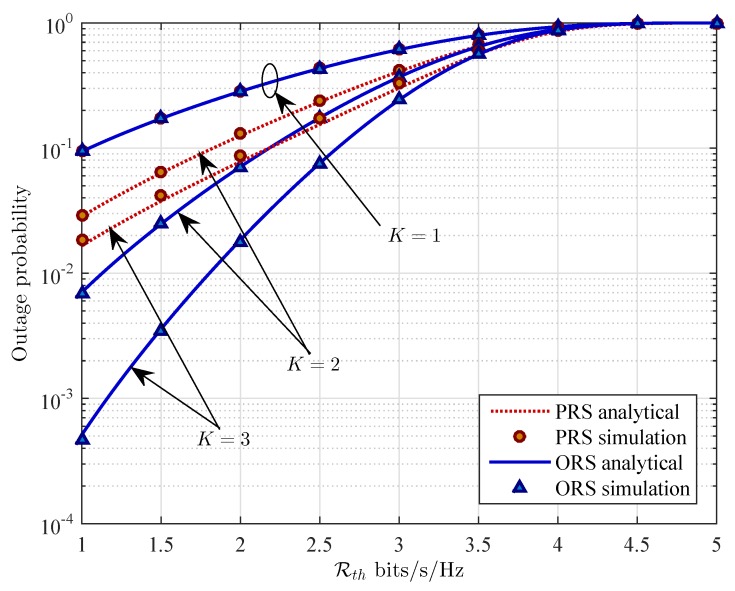
Outage probability $P_{\mathrm{out}}$ *versus* the target data rate $R_{th}$ (bits/z/Hz) for different values of the number of relays *K* with ρ=0.3, and $P_{S}=10$ dB.

In Figure 7, we show the outage probability of the two schemes as a function of the target data rate $R_{th}$. Increasing target data rate $R_{th}$ allows the source and the relays transmit with higher data rate. However, as shown in Figure 7, the OP of the wireless energy harvesting relaying system increases along with the increasing of the target data rate.

From Figure 5 to Figure 7, we can observe that for a given system parameter (e.g., *ρ*, $x_{R}$, or $R_{th}$), the OP of the PRS scheme is higher than that of the ORS scheme. In other words, the ORS scheme outperforms the PRS scheme. As shown in those figures, the different gap of performance gradually decreases as the value of those system parameters increases. On the other hand, based on the slope of the curves in Figure 6 and Figure 7, it is pointed out that the location of relays and the target data rate have more impact on the OP of the ORS scheme than that of the PRS scheme. In addition, the slope of the OP curve of the ORS scheme is larger than that of the PRS scheme. In other words, the ORS scheme achieves lager diversity gain than the PRS scheme does.

We next compare the performance of PRS and ORS schemes in wireless energy harvesting cooperative with those in conventional cooperative networks (*i.e.*, without using energy harvesting at relays) by using simulation results. In conventional cooperative networks, the relays are not powered by using the WEH technology. In stead of that, the relays forward the source signal with a constant transmit power from their own energy. We assume that the source and the relays use the same transmit power level in conventional cooperative networks.

**Figure 8:**
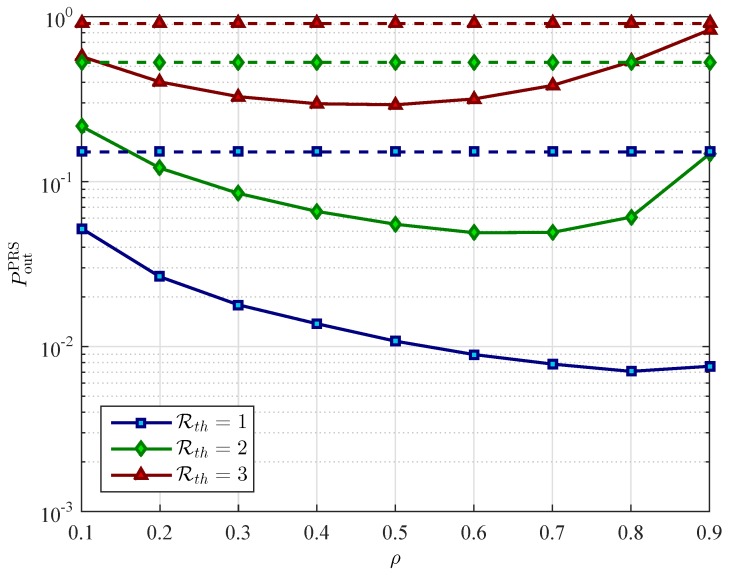
The outage probability (OP) of partial relay selection (PRS) scheme $P_{\mathrm{out}}^{\mathrm{PRS}}$ *versus* the power splitting ratio *ρ* for different values of the target data rate $R_{th}$ with $x_{R}=0.4$, $P_{S}=10$ dB, and K=3 relays. The solid lines represent the OP of the PRS scheme in wireless energy harvesting cooperative networks. The dashed lines represent the OP of the PRS scheme in conventional cooperative networks.

**Figure 9:**
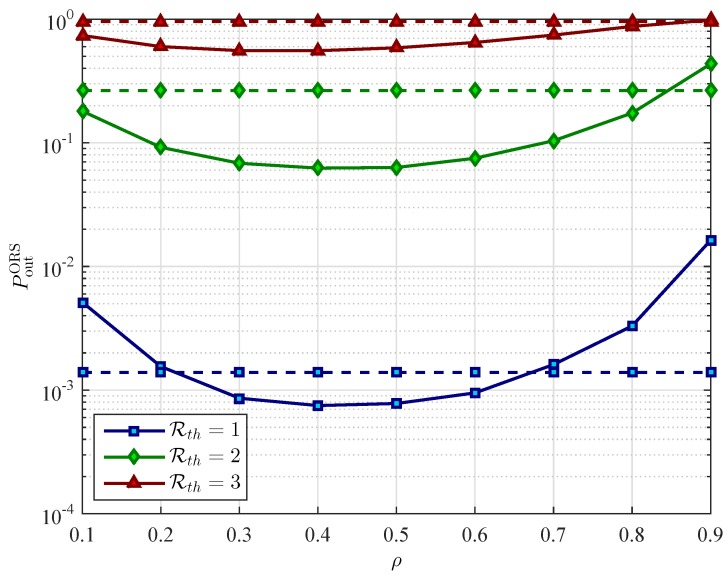
The OP of PRS scheme $P_{\mathrm{out}}^{\mathrm{ORS}}$ *versus* the power splitting ratio *ρ* for different values of the target data rate $R_{th}$ with $P_{S}=10$ dB, and K=3 relays. The solid lines represent the OP of the ORS scheme in wireless energy harvesting cooperative networks. The dashed lines represent the OP of the ORS scheme in conventional cooperative networks.

Figure 8 and Figure 9 present the performance comparisons between the WEH cooperative networks and the conventional cooperative networks in the case of using PRS scheme and ORS scheme, respectively. To capture the effect of using wireless energy harvesting on relay selection, we plot the OP of the two schemes as a function of *ρ* under different value of $R_{th}$. As it can be seen in Figure 8, the PRS scheme with WEH gives better outage performance than without using WEH, especially at low data rate transmission. In contrast to the PRS scheme, the WEH technology just improves the performance of the ORS scheme in some specific ranges of the value of the power splitting ratio *ρ* as can be observer in Figure 9.

**Figure 10:**
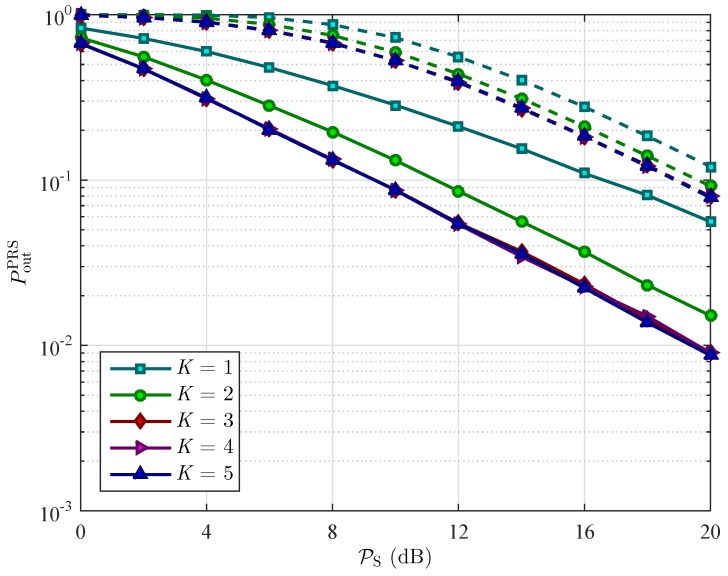
Outage probability $P_{\mathrm{out}}^{\mathrm{PRS}}$ of the PRS scheme *versus* the transmit power of the source $P_{S}$ for different values of the number of relays *K* with ρ=0.3, and $R_{th}=3$ bits/s/Hz. The solid lines represent the OP of the PRS scheme in wireless energy harvesting cooperative networks. The dashed lines represent the OP of the PRS scheme in conventional cooperative networks.

**Figure 11:**
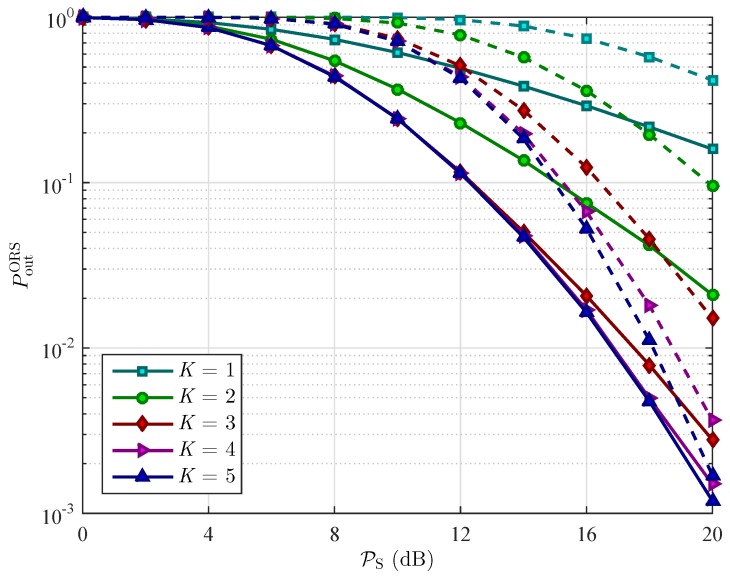
Outage probability $P_{\mathrm{out}}^{\mathrm{ORS}}$ of the PRS scheme *versus* the transmit power of the source $P_{S}$ for different values of the number of relays *K* with ρ=0.3, and $R_{th}=3$ bits/s/Hz. The solid lines represent the OP of the ORS scheme in wireless energy harvesting cooperative networks. The dashed lines represent the OP of the ORS scheme in conventional cooperative networks.

Figure 10 and Figure 11 plot the OP of the two schemes, respectively, as a function of transmit power of the source $P_{S}$ in order to show the diversity gain of the cooperative networks with and without using WEH technology. As shown in Figure 10, when the number of relays increases from K=2 to 5, the OP of PRS scheme with WEH decreases while the the one of PRS scheme without WEH does not vary. The OP of the ORS scheme decreases as the number of relays increases in both WEH and conventional cooperative networks as shown in Figure 11. On the other hand, we can observe that as the transmit power of the source increases, the outage performance in both schemes is better.

## 5. Conclusions

In this paper, we are concerned with the outage performance of a dual-hop DF relaying network where the relays are equipped with wireless energy harvesting technology. On the other hand, relay selection schemes are used in the network in order to increase the diversity gain. We investigated two relay selection schemes: partial relay selection (PRS) and optimal relay selection (ORS) scheme. The PRS scheme selects the relay that will assist the source-destination transmission based only the CSI of the channel from source to the relays, while the ORS scheme takes into account the CSI of both two hops. The system performance is analyzed in terms of outage probability over the independent and non-identical Rayleigh fading channels. We have obtained the closed-form approximation for the outage probabilities of both schemes. The numerical results have shown that the ORS scheme provides better outage performance and larger diversity gain compared to the PRS scheme for the same network setting. However, the ORS scheme increases the cooperative overhead and is more dependent on the relays’ position and the target data rate than the PRS scheme. On the other hand, the PRS scheme provides better outage performance and obtain higher diversity gain in WEH cooperative networks compared to the convention cooperative networks where the WEH technology is not used at the relays.

## Appendix A. Proof of Lemma 1

Using Equation (11), the CDF of $g_{{\mathrm{SR}}_{b}}^{\mathrm{PRS}}$ is given by

(A1) $$\begin{array}{cc} F_{g_{{\mathrm{SR}}_{b}}^{\mathrm{PRS}}} & =\mathrm{Pr}(g_{{\mathrm{SR}}_{b}}^{\mathrm{PRS}}<z)\\ & =\mathrm{Pr}\left(\max_{k\in R}g_{{\mathrm{SR}}_{k}}<z\right)\\ & =\prod_{k=1}^{K}\left(g_{{\mathrm{SR}}_{k}}<z\right) \end{array}$$

The last equal sign in Equation (A1) is due to the fact that the channels are assumed to be independent. On the other hand, $g_{{\mathrm{SR}}_{k}}$ follows exponential distribution with parameter $\lambda_{{\mathrm{SR}}_{k}}$, the CDF of $g_{{\mathrm{SR}}_{b}}^{\mathrm{PRS}}$ is further expressed as

(A2) $$F_{g_{{\mathrm{SR}}_{b}}^{\mathrm{PRS}}}=\prod_{k=1}^{K}\left(1-e^{-\frac{z}{\lambda_{{\mathrm{SR}}_{k}}}}\right)$$

By using the identity [25]

(A3) $$\prod_{k=1}^{K}\left(1-x_{k}\right)=1-\sum_{k=1}^{K}{\left(-1\right)}^{k-1}\underset{n_{1}<\cdots <n_{k}}{\sum_{n_{1}=1}^{K}\cdots \sum_{n_{k}=1}^{K}}\prod_{t=1}^{k}x_{n_{t}},$$

the CDF of $g_{{\mathrm{SR}}_{b}}^{\mathrm{PRS}}$ is obtained as in Equation (15). Taking derivative of Equation (15), we easily obtain the corresponding PDF Equation (16).

## Appdix B. Proof of Corollary 1

Let $x≜g_{{\mathrm{SR}}_{b}}^{\mathrm{PRS}}$. $I_{2}$ conditioned on *x* is obtained as

(B1) $$\begin{array}{cc} I_{2} & =\mathrm{Pr}(\Psi_{R}g_{{\mathrm{SR}}_{b}}^{\mathrm{PRS}}\geq \gamma_{th},\Psi_{D}g_{{\mathrm{SR}}_{b}}^{\mathrm{PRS}}g_{R_{b}D}^{\mathrm{PRS}}<\gamma_{th})\\ & =\mathrm{Pr}\left(g_{R_{b}D}^{\mathrm{PRS}}<\frac{\gamma_{th}}{\Psi_{D}x}|x\geq \frac{\gamma_{th}}{\Psi_{R}}\right) \end{array}$$

By using the total probability theorem, $I_{2}$ is further obtained as

(B2) $$\begin{array}{cc} I_{2} & =\frac{1}{K}\sum_{k=1}^{K}\mathrm{Pr}\left(g_{R_{k}D}<\frac{\gamma_{th}}{\Psi_{D}x}|x\geq \frac{\gamma_{th}}{\Psi_{R}}\right)\\ & =\frac{1}{K}\sum_{k=1}^{K}\int_{\gamma_{th}/\Psi_{R}}^{\infty }F_{g_{R_{k}D}}\left(\frac{\gamma_{th}}{\Psi_{D}x}\right)f_{g_{{\mathrm{SR}}_{b}}^{\mathrm{PRS}}}\left(x\right)dx \end{array}$$

Using Equation (15) in Lemma 1, and after some algebraic manipulations, $I_{2}$ is obtained as

(B3) $$\begin{array}{cc} I_{2} & =\frac{1}{K}\sum_{k=1}^{K}\int_{\gamma_{th}/\Psi_{R}}^{\infty }\left(1-e^{-\frac{\gamma_{th}}{\Psi_{D}\lambda_{R_{k}D}x}}\right)\sum_{l=1}^{K}{\left(-1\right)}^{l-1}\underset{n_{1}<\cdots <n_{l}}{\sum_{n_{1}=1}^{K}\cdots \sum_{n_{l}=1}^{K}}\sum_{t=1}^{l}\frac{1}{\lambda_{{\mathrm{SR}}_{n_{t}}}}e^{-x\sum_{t=1}^{l}\frac{1}{\lambda_{{\mathrm{SR}}_{n_{t}}}}}dx \end{array}$$

With some arrangements, we obtain $I_{2}$ as in Equation (19).

## Appdix C. Proof of Lemma 2

From Equations (4) and (7), the CDF of $\gamma_{e2e,k}$ is expressed as

(C1) $$\begin{array}{cc} F_{\gamma_{e2e,k}}\left(z\right) & =\mathrm{Pr}(\min\left(\gamma_{{\mathrm{SR}}_{k}},\gamma_{R_{k}D}\right)<z)\\ & =\mathrm{Pr}(\min\left(\Psi_{R},\Psi_{D}g_{R_{k}D}\right)g_{{\mathrm{SR}}_{k}}<z)\\ & =\underset{P_{1}}{\underset{︸}{\mathrm{Pr}(\Psi_{R}\leq \Psi_{D}g_{R_{k}D},\Psi_{R}g_{{\mathrm{SR}}_{k}}<z)}}+\underset{P_{2}}{\underset{︸}{\mathrm{Pr}(\Psi_{R}>\Psi_{D}g_{R_{k}D},\Psi_{D}g_{{\mathrm{SR}}_{k}}g_{R_{k}D}<z)}} \end{array}$$

Using Equations (11) and (12), $P_{1}$ is calculated as

(C2) $$\begin{array}{cc} P_{1} & =\mathrm{Pr}\left(g_{R_{k}D}\geq \frac{\Psi_{R}}{\Psi_{D}}\right)\mathrm{Pr}\left(g_{{\mathrm{SR}}_{k}}<\frac{z}{\Psi_{R}}\right)\\ & =\left(1-F_{g_{R_{k}D}}\left(\frac{\Psi_{R}}{\Psi_{D}}\right)\right)F_{g_{{\mathrm{SR}}_{k}}}\left(\frac{z}{\Psi_{R}}\right)\\ & =\left(1-e^{-\frac{z}{\Psi_{R}\lambda_{{\mathrm{SR}}_{k}}}}\right)e^{-\frac{\Psi_{R}}{\Psi_{D}\lambda_{R_{k}D}}} \end{array}$$

By using the conditional probability, $P_{2}$ is calculated as

(C3) $$\begin{array}{cc} P_{2} & =\mathrm{Pr}\left(g_{{\mathrm{SR}}_{k}}<\frac{z}{\Psi_{D}x}|g_{R_{k}D}=x,x<\frac{\Psi_{R}}{\Psi_{D}}\right)\\ & =\int_{0}^{\Psi_{R}/\Psi_{D}}\mathrm{Pr}\left(g_{{\mathrm{SR}}_{k}}<\frac{z}{\Psi_{D}x}\right)f_{g_{R_{k}D}}\left(x\right)dx \end{array}$$

With the helps of Equations (11) and (12), we obtain $P_{2}$ as

(C4) $$\begin{array}{cc} P_{2} & =\int_{0}^{\Psi_{R}/\Psi_{D}}\left(1-e^{-\frac{z}{\Psi_{D}\lambda_{{\mathrm{SR}}_{k}}x}}\right)\frac{1}{\lambda_{R_{k}D}}e^{-\frac{x}{\lambda_{R_{k}D}}}dx\\ & =1-e^{-\frac{\Psi_{R}}{\Psi_{D}\lambda_{R_{k}D}}}-\frac{1}{\lambda_{R_{k}D}}\int_{0}^{\Psi_{R}/\Psi_{D}}e^{-\frac{z}{\Psi_{D}\lambda_{{\mathrm{SR}}_{k}}x}-\frac{x}{\lambda_{R_{k}D}}}dx \end{array}$$

Plugging Equations (C2) and (C4) into Equation (C1), we obtain the CDF of $\gamma_{e2e,k}$ as in Equation (25).

## References

[1] Varshney L. Transporting Information and Energy Simultaneously. Proceedings of the IEEE International Symposium on Information Theory (ISIT).

[2] Krikidis I., Timotheou S., Nikolaou S., Zheng G., Wing D., Ng K., Wing D., Ng K. (2014). Simultaneous Wireless Information and Power Transfer in Modern Communication Systems. IEEE Commun. Mag..

[3] Zhou X., Zhang R., Ho C.K. (2013). Wireless Information and Power Transfer: Architecture Design and Rate-Energy Tradeoff. IEEE Trans. Commun..

[4] Liu P., Gazor S., Kim I., Kim D. (2015). Noncoherent Relaying in Energy Harvesting Communication Systems. IEEE Trans. Wirel. Commun..

[5] Laneman J., Tse D., Wornell G.W. (2004). Cooperative diversity in wireless networks: Efficient protocols and outage behavior. IEEE Trans. Inf. Theory.

[6] Chen H., Liu J., Dong Z., Zhou Y., Guo W. (2011). Exact Capacity Analysis of Partial Relay Selection Under Outdated CSI Over Rayleigh Fading Channels. IEEE Trans. Veh. Technol..

[7] Etezadi F., Zarifi K., Ghrayeb A., Affes S. (2012). Decentralized Relay Selection Schemes in Uniformly Distributed Wireless Sensor Networks. IEEE Trans. Wirel. Commun..

[8] Ulukus S., Yener A., Erkip E., Simeone O., Zorzi M., Grover P., Huang K. (2015). Energy Harvesting Wireless Communications: A Review of Recent Advances. IEEE J. Sel. Areas Commun..

[9] Chen H., Li Y., Luiz Rebelatto J., Uchoa-Filho B., Vucetic B. (2015). Harvest-Then-Cooperate: Wireless-Powered Cooperative Communications. IEEE Trans. Signal Process..

[10] Krikidis I., Timotheou S., Sasaki S. (2012). RF Energy Transfer for Cooperative Networks: Data Relaying or Energy Harvesting?. IEEE Commun. Lett..

[11] Nasir A., Zhou X., Durrani S., Kennedy R. (2013). Relaying Protocols for Wireless Energy Harvesting and Information Processing. IEEE Trans. Wirel. Commun..

[12] Nasir A., Zhou X., Durrani S., Kennedy R. Throughput and ergodic capacity of wireless energy harvesting based DF relaying network. Proceedings of the 2014 IEEE International Conference on Communications (ICC).

[13] Zhong C., Suraweera H., Zheng G., Krikidis I., Zhang Z. (2014). Wireless Information and Power Transfer With Full Duplex Relaying. IEEE Trans. Commun..

[14] Do N.T., Bao V.N.Q., An B. A Relay Selection Protocol for Wireless Energy Harvesting Relay Networks. Proceedings of the 2015 IEEE International Conference on Advanced Technologies for Communications (ATC).

[15] Tang L., Zhang X., Wang X. (2014). Joint Data and Energy Transmission in a Two-Hop Network with Multiple Relays. IEEE Commun. Lett..

[16] Ding Z., Krikidis I., Sharif B., Poor H. (2014). Wireless Information and Power Transfer in Cooperative Networks With Spatially Random Relays. IEEE Trans. Wirel. Commun..

[17] Son P.N., Kong H.Y. (2015). Exact Outage Analysis of Energy Harvesting Underlay Cooperative Cognitive Networks. IEICE Trans. Commun..

[18] Van-Dinh N., Son D.-V., Oh-Soon S. Opportunistic relaying with wireless energy harvesting in a cognitive radio system. Proceedings of the 2015 IEEE Wireless Communications and Networking Conference (WCNC).

[19] Krikidis I. (2015). Relay Selection in Wireless Powered Cooperative Networks With Energy Storage. IEEE J. Sel. Areas Commun..

[20] Michalopoulos D.S., Suraweera H.A., Karagiannidis G.K., Schober R. Amplify-and-Forward Relay Selection with Outdated Channel State Information. Proeedings of the 2010 IEEE Global Telecommunications Conference (GLOBECOM 2010).

[21] Quek T.Q.S., Shin H., Win M.Z. (2007). Robust Wireless Relay Networks: Slow Power Allocation with Guaranteed QoS. IEEE J. Sel. Top. Signal Process..

[22] Zhang R., Ho C.K. (2013). MIMO Broadcasting for Simultaneous Wireless Information and Power Transfer. IEEE Trans. Wirel. Commun..

[23] Bao V.N.Q., Duong T., Benevides da Costa D., Alexandropoulos G., Nallanathan A. (2013). Cognitive Amplify-and-Forward Relaying with Best Relay Selection in Non-Identical Rayleigh Fading. IEEE Commun. Lett..

[24] Gradshteyn I.S., Ryzhik I.M. (2007). Tables of Integrals, Series, and Products.

[25] Duong T., Bao V.N.Q., Zepernick H. (2009). On the performance of selection decode-and-forward relay networks over Nakagami-m fading channels. IEEE Commun. Lett..

